# A promising vaccine candidate against COVID-19

**DOI:** 10.1186/s43556-020-00008-x

**Published:** 2020-09-30

**Authors:** Xiwei Wang, Wenwei Tu

**Affiliations:** Laboratory for Medical Translational Immunology, Department of Paediatrics and Adolescent Medicine, Li Ka Shing Faculty of Medicine, The University of Hong Kong, Hong Kong, SAR China

In a recent study published in (Nature_10.1038/s41586-020-2599-8: 2020), Yang et al. developed a COVID-19 vaccine with high efficacy to induce neutralizing antibodies targeting the receptor-binding domain (RBD) of spike (S) protein (S-RBD) and prevent SARS-CoV-2 infection in multiple animal models, including non-human primates. This study not only gave new insights for developing COVID-19 vaccine, but also provided a readily applicable strategy to prevent SARS-CoV-2 infection.

Severe acute respiratory syndrome coronavirus 2 (SARS-CoV-2) has been spreading worldwide and become a serious global health problem by causing the novel coronavirus disease 2019 (COVID-19). However, no licensed preventative vaccines are available for COVID-19 until now. SARS-CoV-2 contains four structural proteins, including the nucleocapsid, membrane, envelope and S proteins [1]. Among them, S protein is the most important therapeutic target as it plays pivotal roles in viral attachment, entry and fusion. The S protein contains S1 and S2 subunits. The S1 subunit binds to host angiotensin-converting enzyme 2 (ACE2) receptor and mediates viral entry, while the S2 subunit mediates the fusion of virus and host cell membrane [1]. Currently, most vaccine candidates against COVID-19 are designed to target the S protein to elicit neutralizing antibodies for blocking virus entry [2, 3]. Compared with those targeting the whole S protein, the vaccine candidates targeting the S-RBD are more scientifically feasible, because they can avoid inducing the non-neutralizing antibodies targeting the non-RBD regions, which may cause side effects like antigen-dependent enhancement [3, 4]. In addition, the S-RBD region has multiple conformational neutralizing epitopes and is relatively conserved, making it as one of the critical targets for vaccine development against COVID-19.

To develop a COVID-19 vaccine with global supply scale, Yang et al. produced a recombinant S-RBD protein using a commercial baculovirus expression system, which has already been applied to produce many other vaccines, including influenza vaccines [5]. This recombinant S-RBD protein is as small as 34 kDa and has purity over 98%. Although some glycosylation sites were identified on the S-RBD protein, mapping of the SARS-CoV-2, S-RBD and ACE2 complex suggested that these glycosylation sites were far away from the binding sites and probably have few negative impacts on receptor recognition and/or interaction. More importantly, this S-RBD protein has good native conformation and can bind to ACE2 with high affinity, indicating that it is a good vaccine immunogen to induce neutralizing antibodies and prevent viral entry.

Considering aluminum (alum) is a safe and effective vaccine adjuvant, the authors developed the vaccine regimen by co-precipitating alum and the S-RBD protein [5]. All the animals (mice, rabbits and non-human primates) had strong antibody responses after immunization with the alum-precipitated S-RBD vaccine. The S-RBD-specific antibodies could be detectable as early as 7 days post-vaccination in a dose-dependent manner. Interestingly, the S-RBD protein alone also induced specific antibody responses, while addition of alum as the adjuvant promoted the specific immune responses. The elicited specific antibody response was very strong because a positive antibody binding reaction of vaccinated mice sera could still be identified at dilution of 1:204,800. Furthermore, the sera from immunized mice, rabbits or non-human primates had strong viral neutralizing abilities to prevent the ACE2-expressing cells from pseudovirus or live SARS-CoV-2 infection. Importantly, the murine sera collected at 7 days after a single dose vaccination was effective to block S-RBD/ACE2 binding. These data suggested that the S-RBD vaccine could induce abundant neutralizing antibodies and had a great potential to prevent SARS-CoV-2 infection in vivo.

Subsequently, the authors found that the S-RBD vaccine could induce neutralizing antibodies and effectively protect non-human primates from live SARS-CoV-2 infection. Once vaccinated, undetectable or decreased viral genomic and subgenomic RNAs in respiratory system, and no significant histopathological changes in lung tissues were found in SARS-CoV-2-infected non-human primates. These data indicated that the S-RBD vaccine not only controlled viral replication, but also prevented lung pathogenesis in non-human primates [5]. As non-human primates are the closest species to humans by sharing many similarities in physiological, biological and immunological characteristics, the efficacy of the S-RBD vaccine demonstrated in non-human primates is probably reproducible in human beings.

Elevated T cell responses were also found in the mice vaccinated with the S-RBD vaccine. Thus, the authors further evaluated the involvement of cellular immune response in the prevention of SARS-CoV-2 infection [5]. Interestingly, adoptive transferring the sera obtained from vaccinated mice effectively protected the recipient mice from live SARS-CoV-2 infection, while the splenic T cells from same vaccinated mice did not show similar protective effect. Therefore, the protection was mainly mediated by the specific humoral responses induced by the S-RBD vaccine.

Finally, the authors determined the safety profile of the S-RBD vaccine [5]. There was no acceleration of pneumonia or antibody-dependent enhancement in the mice receiving the S-RBD-vaccinated immune sera. Systematic inflammation was not observed either, because no inflammatory cytokines were significantly increased after vaccination. Furthermore, no general symptoms, histological alterations in organs and changes in peripheral blood cells were observed in vaccinated non-human primates. Taken together, the S-RBD vaccine has a good safety profile.

In summary, Yang et al. developed a promising COVID-19 vaccine candidate by targeting the S-RBD of SARS-CoV-2. The vaccine candidate was safe and could effectively induce neutralizing antibodies to protect animals, including non-human primates, from live SARS-CoV-2 infection. As this vaccine candidate can be produced by a commercially feasible system at a massive scale, it has a great potential to fight against the global pandemic of COVID-19 and deserves to be evaluated in clinical trials as soon as possible (Fig. 1).

**Fig. 1:**
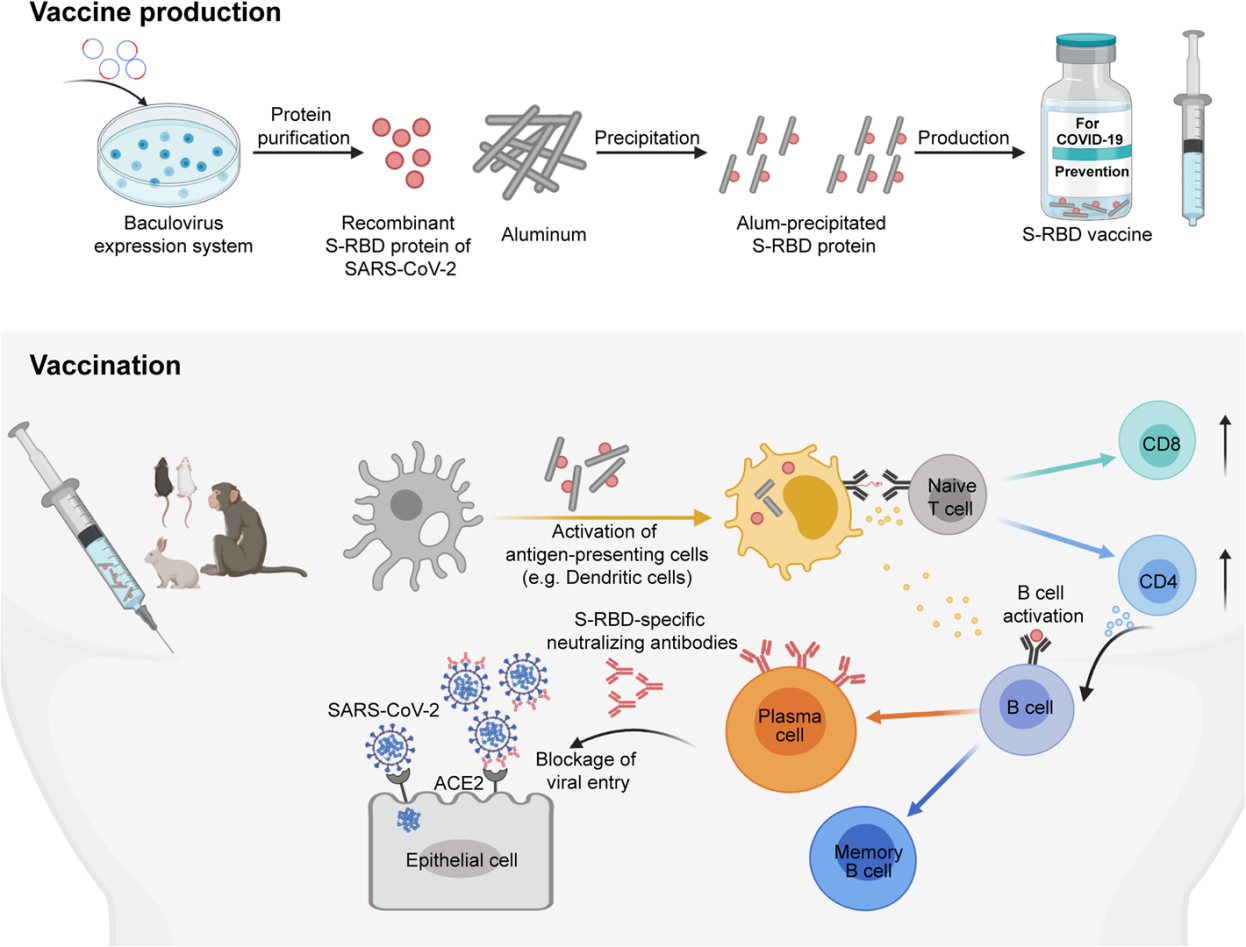
Scheme of generating and evaluating the vaccine candidate targeting the spike protein receptor binding domain (S-RBD) of SARS-CoV-2. The recombinant S-RBD protein comprising residues 319–545 was generated at a massive scale using a Bac-to-Bac baculovirus expression system, followed by precipitation with aluminum (alum). The alum-precipitated S-RBD vaccine was then administrated into mice, rabbits or non-human primates. The vaccine components are taken by antigen-presenting cells (APCs), and then induce the activation and maturation of APCs. The activated APCs prime and drive naïve T cells differentiation into effector cells, including CD4 helper and CD8 T cells. Meanwhile, the resting B cells directly recognize the S-RBD protein and then differentiate into antigen-specific plasma cells and memory B cells under the cytokine support from APCs and helper T cells. The S-RBD vaccine can induce abundant S-RBD-specific neutralizing antibodies secreted from the plasma cells and effectively prevent live SARS-CoV-2 infection by blocking viral entry

## Data Availability

Not applicable.

## Abbreviations

COVID-19: Coronavirus disease 2019
RBD: Receptor-binding domain
SARS-CoV-2: Severe acute respiratory syndrome coronavirus 2
S: Spike
ACE2: Angiotensin-converting enzyme 2
Alum: Aluminum
